# Ultrafast Photochemical Reaction Dynamics of a Cyclic (Alkyl)(Amino)Carbene-Carbon Disulfide Dimer Probed by Femtosecond Infrared Spectroscopy

**DOI:** 10.3390/ijms27167190

**Published:** 2026-08-11

**Authors:** Seongbeom Jeon, Juhyang Shin, Jaegeum Cha, Youngsuk Kim, Manho Lim

**Affiliations:** 1 Department of Chemistry, Pusan National University, Busan 46241, Republic of Korea; s_b.21@pusan.ac.kr (S.J.); dksvmflzk@gmail.com (J.S.); jgjg99@pusan.ac.kr (J.C.); 2 Chemistry Institute for Functional Materials, Pusan National University, Busan 46241, Republic of Korea; 3 Institute for Future Earth, Pusan National University, Busan 46241, Republic of Korea

**Keywords:** reaction dynamics in solution, photodissociation of 1,2,3,4-tetrathiane, femtosecond vibrational spectroscopy, homolytic cleavage of disulfide bonds, stepwise dissociation of two S–S bonds

## Abstract

The ultrafast photochemical reaction dynamics of a cyclic(alkyl)(amino)carbene–carbon disulfide (CAAC–CS_2_) dimer containing two adjacent S–S bonds were investigated using femtosecond time-resolved infrared spectroscopy in combination with multireference electronic structure calculations. Time-resolved vibrational spectra and global kinetic analysis reveal that photoexcitation of the S–S n → σ* transition at 375 nm induces subpicosecond (<0.3 ps) homolytic cleavage of one S–S bond, generating a bis-thiyl diradical intermediate. This intermediate undergoes two competing pathways: recombination to regenerate the parent dimer with a time constant of 5.7–8.5 ps, or secondary cleavage of the remaining S–S bond to yield two CAAC–CS_2_ monomers with a time constant of 30–35 ps. Wavelength- and temperature-dependent kinetic measurements demonstrate that the branching between these pathways is governed by excess excitation energy and thermally driven radical-pair fluctuations. Multireference electronic structure calculations support a sequential S–S bond cleavage mechanism, in good agreement with the experimental observations. These findings provide direct spectroscopic evidence for a bis-thiyl diradical intermediate and offer new mechanistic insight into the ultrafast photochemistry of adjacent S–S bonds.

## 1. Introduction

Stable singlet carbenes have emerged as versatile platforms for stabilizing reactive intermediates and enabling unusual bonding motifs. In particular, cyclic(alkyl)(amino)carbenes (CAACs) exhibit distinctive electronic properties stemming from their strong σ-donor and π-acceptor character, which enables stabilization of radical species and low-valent main-group compounds. These attributes have driven broad applications in catalysis, organometallic chemistry, and small-molecule activation [1,2,3,4,5]. Since the first isolation of stable CAACs, their ambiphilic electronic structure and readily tunable steric profile have distinguished them from conventional N-heterocyclic carbenes [6,7,8,9]. These features have been exploited in transition-metal catalysis, most notably in olefin metathesis, where CAAC ligands form robust metal-carbene bonds and can improve catalyst longevity and productivity [10,11], as well as in a broader range of metal-catalyzed transformations [12]. Stable carbenes can also mimic aspects of transition-metal reactivity in small-molecule activation and stabilize low-coordinate or open-shell main-group and transition-metal species [7,8,9].

The CAAC–CS_2_ system extends these advantages into sulfur-rich and redox-active molecular chemistry. Reactions of stable carbenes with heteroallenes such as CS_2_ generate dithiocarboxylate or carbene-stabilized CS_2_ adducts whose structures and bonding provide sensitive probes of carbene stereoelectronics [13]. CAAC-derived CS_2_ frameworks are particularly attractive because the CAAC scaffold can stabilize sulfur-centered radical character and sulfur-rich architectures [14,15]. In this context, the CAAC–CS_2_ monomer is especially notable because it undergoes thermally induced cyclodimerization to form a tetrathiane structure containing two adjacent S–S bonds, whereas photoirradiation reverses this transformation to regenerate the monomer [16]. This reagent-free, stimulus-dependent, and reversible interconversion provides a promising platform for stimuli-responsive molecular systems based on reversible S–S bond formation and cleavage. However, realizing such control requires a detailed understanding of the photochemical dynamics of dual S–S bonds within a single molecule.

Photochemical cleavage of S–S bonds plays a central role in sulfur-centered radical chemistry and stimuli-responsive materials [17,18,19,20]. Although ultrafast photodissociation of single disulfide bonds via n → σ* excitation is well established, producing two thiyl radicals through homolytic S–S bond cleavage [17,21,22,23], the reaction dynamics of systems containing dual S–S bonds remain poorly understood. In particular, it is unclear whether the two S–S bonds cleave concertedly or sequentially, nor how the short-lived species formed immediately after excitation evolve to determine the final outcome. The CAAC–CS_2_ dimer provides an ideal model for addressing these questions because it contains two adjacent S–S bonds that may undergo coupled or sequential dissociation. Electronic excitation into the sulfur lone-pair to σ* manifold is expected to weaken these bonds and initiate fragmentation; however, the detailed mechanism and the identities of the transient species remain unresolved. Femtosecond time-resolved infrared (TRIR) spectroscopy is well suited to interrogate such processes by tracking structural evolution through vibrational signatures. Unlike transient electronic spectroscopy, TRIR enables more definitive assignment of intermediates via diagnostic vibrational modes [24,25]. In the CAAC–CS_2_ system, the dimer and monomer display distinct vibrational features, enabling real-time discrimination among reactant, product, and intermediate species.

Here, we employ femtosecond TRIR spectroscopy combined with multireference electronic structure calculations to investigate the solution-phase photochemical reaction dynamics of the CAAC–CS_2_ dimer. The results reveal subpicosecond homolytic cleavage of one S–S bond to generate a bis-thiyl diradical (DR) intermediate with vibrational features distinct from those of both the dimer and the monomer. Time-resolved spectral and kinetic analyses further demonstrate that the DR proceeds through competing pathways leading to either dimer regeneration or monomer formation, with kinetics that depend on both the excitation wavelength and the temperature. Together, these results provide direct experimental evidence for a DR intermediate and support a stepwise mechanism for dual S–S bond cleavage, offering new mechanistic insight into the ultrafast photochemistry of adjacent S–S bonds.

## 2. Results and Discussion

**Figure 1:**
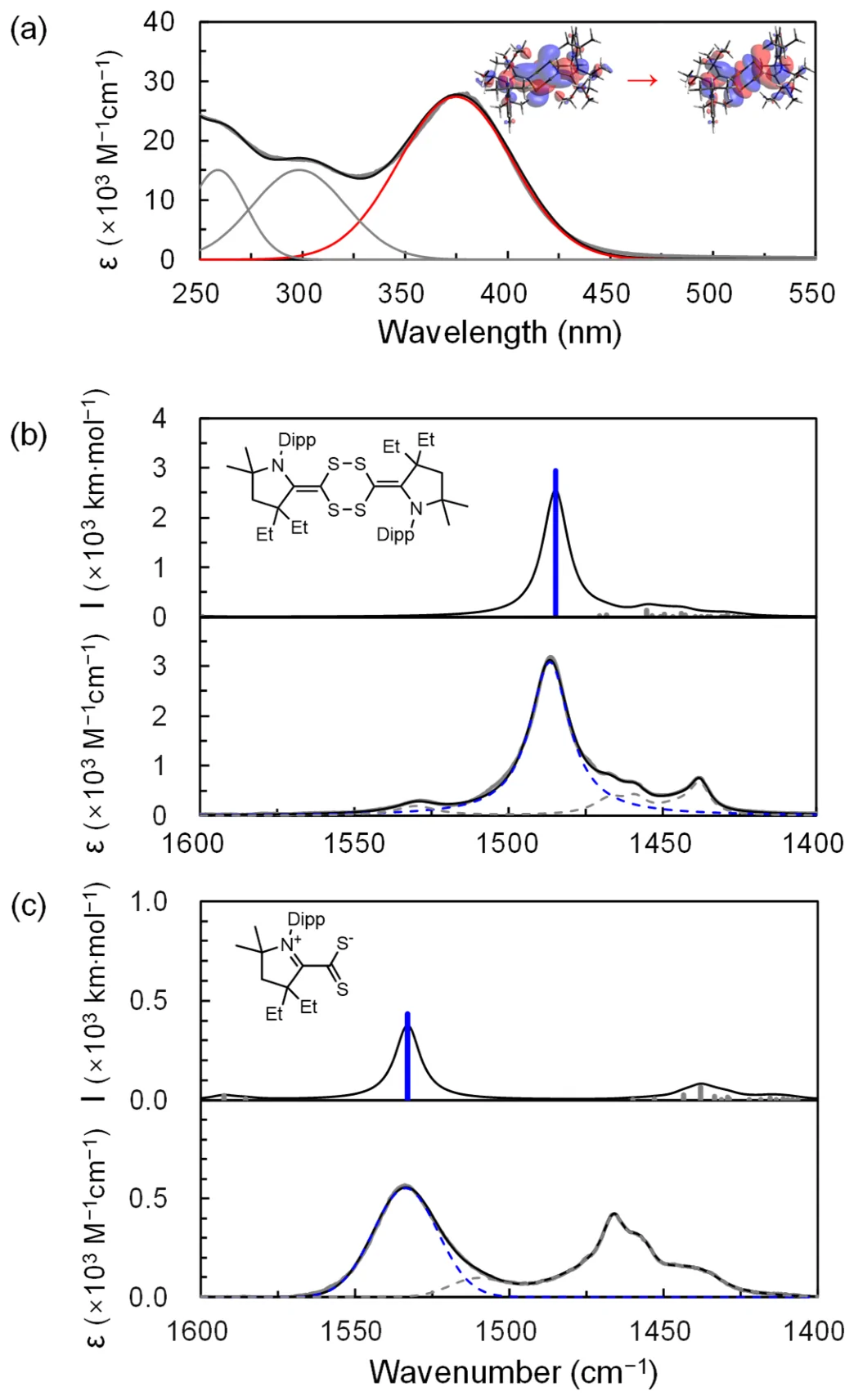
(**a**) Steady-state UV–vis absorption spectrum of the CAAC–CS_2_ dimer in CD_2_Cl_2_ at room temperature. Three absorption bands are observed in the 250–500 nm region. The lowest-energy band, centered at 375 nm (red line), is assigned to the S–S n → σ* transition; the corresponding CASSCF active orbitals involved in this excitation are also shown. Steady-state FTIR absorption spectra of (**b**) the CAAC–CS_2_ dimer and (**c**) the CAAC–CS_2_ monomer in CD_2_Cl_2_ at room temperature are presented in the lower panels, together with their molecular structures. The corresponding DFT-calculated vibrational spectra are shown in the upper panels. The calculated vibrational frequencies were scaled by factors of 0.980 and 0.969 for the CAAC–CS_2_ dimer and monomer, respectively. The simulated spectra were generated by applying line broadening to the calculated stick spectra. Note that the intensity scale of the *y*-axis in (**c**) is fourfold smaller than that in (**b**). For the experimental spectra, the *y*-axis is shown as the extinction coefficient (ε), obtained by dividing the absorbance by the sample concentration and the pathlength of the sample cell. For the calculated IR spectra, the *y*-axis is shown as the calculated IR intensity (I). Experimental UV-vis spectrum (thick gray line) was reproduced by the sum (black line) of three bands (thin gray and red lines). Experimental IR spectra (thick gray lines) were well reproduced by the sum (black lines) of several bands (dashed lines), and the color of each dashed band matches the assigned stick(s) in the calculated spectra.

Figure 1a shows the electronic absorption spectrum of the CAAC–CS_2_ dimer in CD_2_Cl_2_ solution, deconvoluted into three bands spanning 250–500 nm. The lowest-energy band, peaking at 375 nm, is assigned to a sulfur n → S–S σ* transition to the S_1_ state, which promotes dimer dissociation. This assignment is supported by time-dependent density functional theory (TD-DFT, B3LYP-GD3/def2-SVP [26,27]) and complete active space self-consistent field (CASSCF/cc-pVDZ [28]) calculations. For the TRIR experiments, excitation at 350 or 400 nm (on either side of the 375 nm band) was used to probe the photodissociation dynamics of the CAAC–CS_2_ dimer. Figure 1b,c show the equilibrium Fourier transform infrared (FTIR) spectra of the CAAC–CS_2_ dimer and CAAC–CS_2_ monomer in CD_2_Cl_2_ solution, respectively, in the 1600–1400 cm^−1^ region. Vibrational assignments were guided by DFT calculations at the B3LYP-GD3/def2-SVP level (Table S1) [26,27]. For the CAAC–CS_2_ dimer, the dominant band at 1486 cm^−1^ is assigned to the C=C stretching mode. In contrast, the CAAC–CS_2_ monomer exhibits substantially weaker bands at 1533 and 1466 cm^−1^, assigned to the C=N stretching and CH_2_ bending modes, respectively. These diagnostic bands were used as spectral markers in the TRIR analysis. The optimized equilibrium structures of the CAAC–CS_2_ dimer and CAAC–CS_2_ monomer indicate pronounced structural differences. The CAAC–CS_2_ dimer adopts a nearly planar geometry with an N–C–C–S torsion angle of 16.1°, whereas the CAAC–CS_2_ monomer is nonplanar, with an N–C–C–S torsion angle of 90.5°. These geometries are consistent with reported X-ray crystal structures, which give N–C–C–S torsion angles of 12° for the CAAC–CS_2_ dimer and 92° for the CAAC–CS_2_ monomer [16,29]. This structural distinction is reflected in the vibrational spectra: the C=C stretching mode is characteristic of the CAAC–CS_2_ dimer, whereas the C=N stretching mode is observed for the CAAC–CS_2_ monomer.

**Figure 2:**
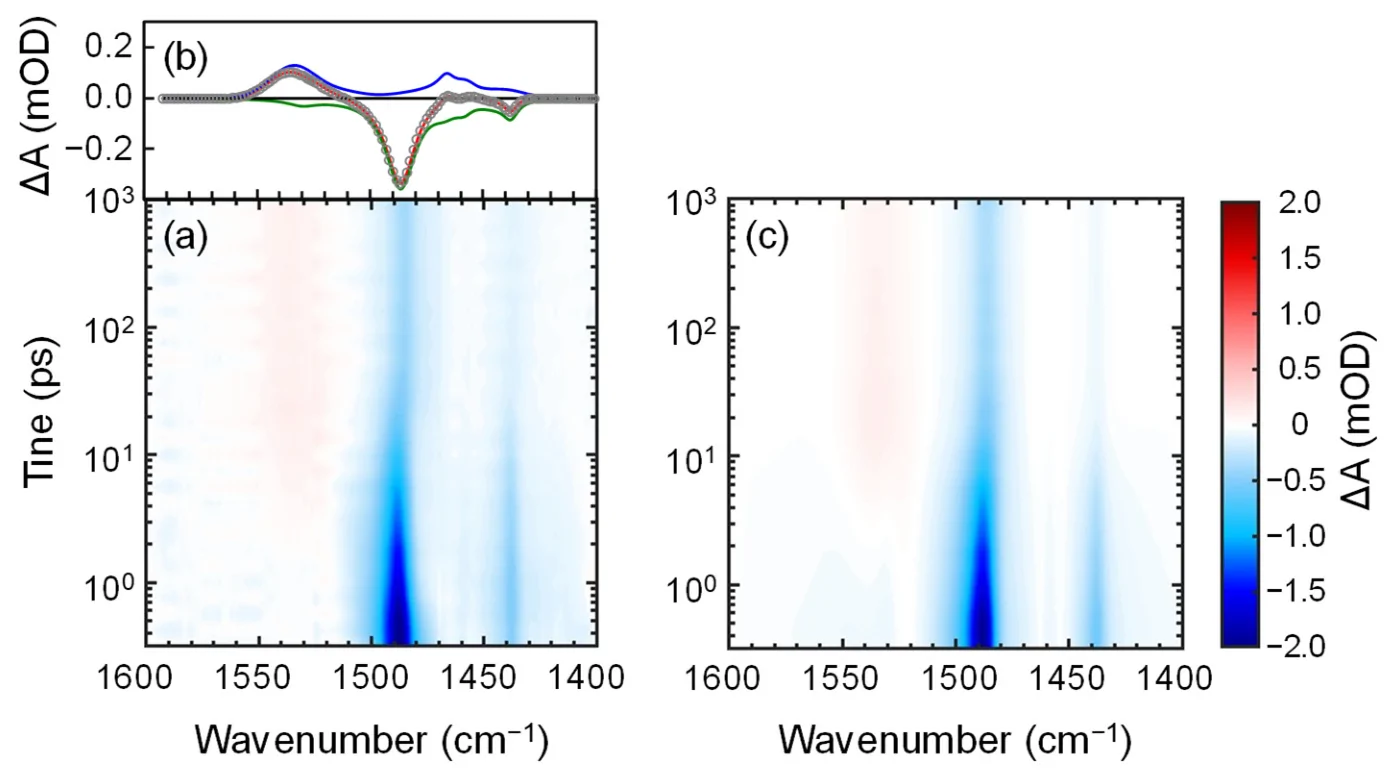
(**a**) Two-dimensional contour map of femtosecond TRIR spectra of the CAAC–CS_2_ dimer in CD_2_Cl_2_ at 293 K following excitation at 400 nm. (**b**) Averaged TRIR spectrum over the 100–1000 ps time window (open circles), reproduced by a linear combination (red line) of the equilibrium spectrum of CAAC–CS_2_ (blue line) and the inverted equilibrium spectrum of the CAAC–CS_2_ dimer (green line). The black line indicates the baseline at ΔA = 0. (**c**) Two-dimensional contour map of the TRIR spectra reconstructed by global fitting using the three basis spectra shown in Figure 3a.

Femtosecond TRIR spectra of the CAAC–CS_2_ dimer in CD_2_Cl_2_ at 293 K were collected in the 1600–1400 cm^−1^ region over delay times of 0.3–1000 ps after 400 nm excitation. Figure 2a presents a two-dimensional contour map of the TRIR spectra. Negative-going features (bleaches) at the frequencies of the equilibrium CAAC–CS_2_ dimer bands arise from depletion of the ground-state reactant population. The prompt appearance of the bleach indicates that photoexcitation and/or photodissociation occurs within 0.3 ps, the earliest delay time accessible in the present experiment. The bleach signal decays on the picosecond timescale, while a new absorption band centered at 1533 cm^−1^ emerges within a few picoseconds and persists, indicating the formation of CAAC–CS_2_. Because the spectral evolution is essentially complete within several tens of picoseconds, the TRIR spectra from 100 to 1000 ps were averaged to facilitate identification of the spectral components at the final stage of the photoreaction. The averaged spectrum is well reproduced by a linear combination of the equilibrium spectrum of CAAC–CS_2_ and the inverted equilibrium spectrum of the CAAC–CS_2_ dimer (Figure 2b). In this fit, the ratio of the integrated area of the CAAC–CS_2_ contribution to that of the CAAC–CS_2_ dimer contribution is 0.60, which is twice the ratio of their integrated extinction coefficients in the 1600–1400 cm^−1^ region. This agreement supports complete dissociation of one CAAC–CS_2_ dimer into two CAAC–CS_2_ monomers.

**Figure 3:**
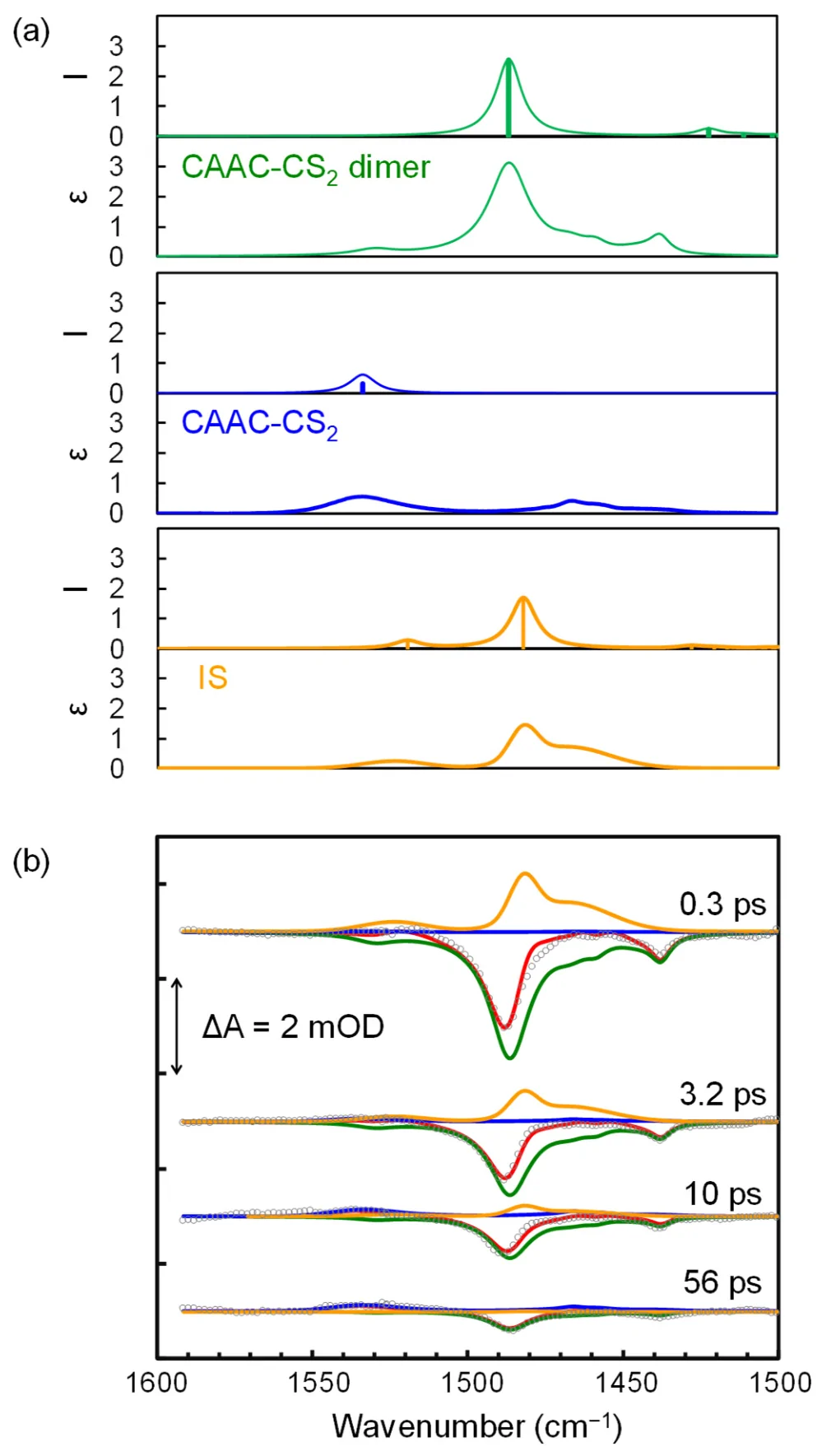
(**a**) Basis spectra used for the global analysis, together with the corresponding DFT-calculated vibrational spectra of the truncated model compounds (see text). The basis spectra for the CAAC–CS_2_ dimer and CAAC–CS_2_ correspond to their equilibrium FTIR spectra, whereas that of the intermediate species (IS) was extracted from the global analysis of the TRIR dataset. The calculated vibrational frequencies were scaled by factors of 0.960, 0.918, and 0.959 for the CAAC–CS_2_ dimer, CAAC–CS_2_, and IS, respectively. The simulated spectra were generated by applying line broadening to the calculated stick spectra, which are shown alongside the corresponding basis spectra. For the experimental spectra, the *y*-axis is shown as the extinction coefficient (ε, ×10^3^ M^−1^cm^−1^), obtained by dividing the absorbance by the sample concentration and the pathlength of the sample cell. For the calculated spectra, the *y*-axis is shown as the calculated IR intensity (I, ×10^3^ km·mol^−1^). (**b**) Representative TRIR spectra of the CAAC–CS_2_ dimer in CD_2_Cl_2_ at 293 K following 400 nm excitation. The experimental spectra (open circles) are well reproduced by a linear combination (red lines) of the three basis spectra: CAAC–CS_2_ dimer (green), CAAC–CS_2_ (blue), and IS (orange), shown in (**a**).

The early-time TRIR spectra (<100 ps) cannot be reproduced by a linear combination of the dimer and monomer spectra alone, indicating the presence of an additional spectral component. This component appears concurrently with the formation of the CAAC–CS_2_ dimer bleach but before the emergence of the characteristic vibrational bands of the monomer. It decays on a timescale of a few picoseconds, with kinetics correlated with both the rise of the CAAC–CS_2_ monomer signal and the recovery of the CAAC–CS_2_ dimer bleach. Accordingly, in addition to the equilibrium spectra of the CAAC–CS_2_ dimer and CAAC–CS_2_, an additional spectrum was included to reproduce the TRIR data (see Figure 3). The entire TRIR dataset was successfully globally fitted using three spectra (hereafter, basis spectra) shown in Figure 3a. The additional spectrum more closely resembles that of the CAAC–CS_2_ dimer than that of CAAC–CS_2_, and exhibits a prominent band at 1482 cm^−1^. As shown in Figure 2c and Figure 3b, the TRIR spectra are well reproduced by a linear combination of these three basis spectra.

**Figure 4:**
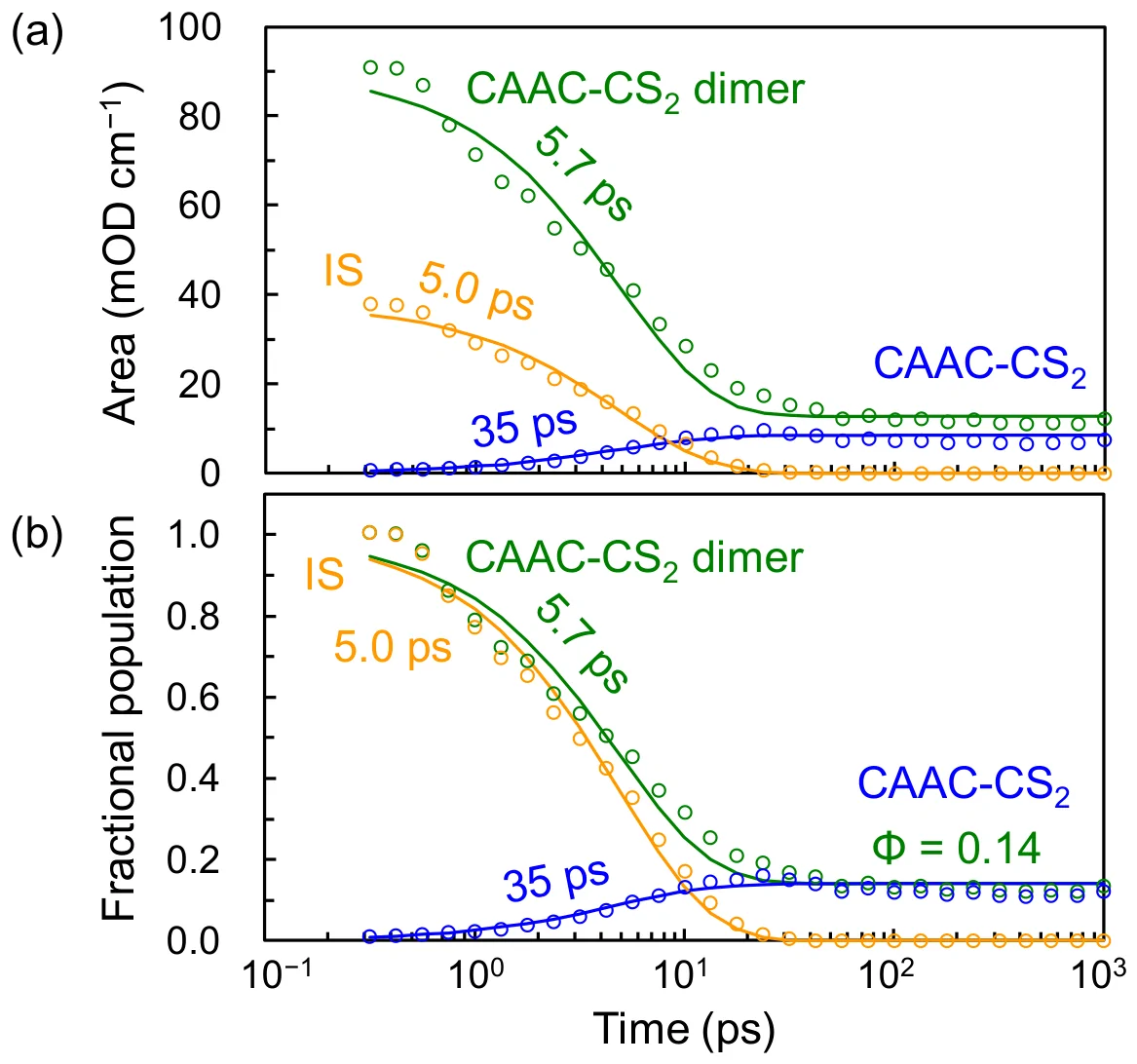
(**a**) Time-dependent changes in the integrated areas of the three basis spectra used to reproduce the TRIR spectra. The integrated areas of the CAAC–CS_2_ and IS represent their absorption signals, whereas that of the CAAC–CS_2_ dimer corresponds to the ground-state bleach amplitude. The experimental data (open circles) are well described by single-exponential fits (solid lines), and the corresponding time constants are indicated. (**b**) Fractional population dynamics of the species represented by the three basis spectra. The populations were obtained by normalizing the time-dependent integrated spectral areas to the integrated extinction coefficients of the corresponding spectra. The CAAC–CS_2_ dimer trace represents the depleted ground-state population, whereas the IS and CAAC–CS_2_ traces represent their respective populations. The quantum yield (Φ) for dimer dissociation is also shown.

The time-dependent area changes in the three basis spectra obtained from the global fit are presented in Figure 4a. The amplitude of the CAAC–CS_2_ signal increases with a time constant of 35 ps, whereas the CAAC–CS_2_ dimer signal recovers with a time constant of 5.7 ps, accompanied by decay of the additional component with a time constant of 5.0 ps. Based on these dynamics, the additional spectrum is assigned to an intermediate species (IS). IS forms within 0.3 ps and subsequently partitions between recombination to regenerate the CAAC–CS_2_ dimer and dissociation to yield CAAC–CS_2_. The population dynamics of the species involved (Figure 4b) were obtained by accounting for the integrated extinction coefficients of the equilibrium spectra of the CAAC–CS_2_ dimer and CAAC–CS_2_ and by assuming that the integrated extinction coefficient of IS is 0.40 times that of the dimer, which provided the best kinetic consistency. This value was selected to best reproduce the observed time-dependent area changes (Figure S1).

The finding that IS formation coincides with depletion of the dimer population, and that IS decay is temporally correlated with both growth of the CAAC–CS_2_ monomer signal and recovery of the CAAC–CS_2_ dimer signal, suggests that IS corresponds either to (i) an electronically excited state of the CAAC–CS_2_ dimer (excited dimer, ED) or (ii) an intermediate formed after the initial homolytic bond-cleavage event but before full dissociation into two stable monomers along the reaction coordinate. Accordingly, as shown in Figure S2, two kinetic schemes were proposed to describe the population dynamics: in Scheme I, IS is an ED; in Scheme II, IS is either a bis-thiyl DR formed by homolytic cleavage of one of the two S–S bonds or a CAAC–CS_2_ pair produced by homolytic cleavage of both S–S bonds, yielding a solvent-caged geminate pair (GP) with incomplete separation. In Scheme I, photoexcitation produces ED within 0.3 ps, which either relaxes to the ground state with a time constant of 5.7 ps or dissociates into two stable monomers with a time constant of 35 ps. Conversely, Scheme II involves prompt S–S bond cleavage (<0.3 ps), followed by either a stepwise or concerted pathway. In the stepwise pathway, homolytic cleavage of the first S–S bond yields a bis-thiyl DR, which either recombines to regenerate the dimer with a time constant of 5.7 ps or undergoes secondary S–S bond cleavage to form stable monomers with a time constant of 35 ps. In the concerted pathway, simultaneous cleavage of both S–S bonds generates a GP with incomplete spatial separation, because the solvent cage can confine the fragments and sustain close intermolecular interactions. This GP either undergoes geminate recombination to reform the dimer with a time constant of 5.7 ps or escapes the solvent cage to yield stable monomers with a time constant of 35 ps. As noted above, the IS spectrum, with a dominant band at 1482 cm^−1^, closely resembles that of the dimer (see Figure 3a) but differs markedly from the monomer spectrum expected for concerted S–S bond cleavage. Calculated vibrational frequencies for a bis-thiyl DR, obtained at the B3LYP-GD3/def2-SVP level using a truncated model compound in which the Dipp and ethyl substituents were replaced by methyl groups, predict a red-shifted, less intense C=C stretching band, in good agreement with the observed IS spectrum (Figure 3a and Table S2). These spectral features strongly suggest that IS corresponds to a DR rather than a dissociated GP. However, the IS spectrum alone cannot unambiguously distinguish ED from DR, because the vibrational frequencies of ED are unknown and not readily accessible by calculation. The secondary dissociation rate of DR is expected to depend on the excess energy of the nascent photoproducts as well as on temperature. In contrast, the dissociation and electronic relaxation rates of ED are not expected to be sensitive to temperature or excitation wavelength (λ_ex_), which influences the excess energy of the nascent photoproducts, provided that both excitation wavelength access the same electronic state. To distinguish between these two schemes, additional experiments were performed in which λ_ex_ and temperature were varied. TRIR spectra of the CAAC–CS_2_ dimer in CD_2_Cl_2_ were recorded in the 1600–1400 cm^−1^ region over 0.3–1000 ps following excitation at 350 nm (λ_ex_ variation) at 293 K, and at 273 K following excitation at 350 nm (temperature variation). Both datasets were analyzed using the same procedure applied to the data collected at 400 nm and 293 K. Two-dimensional contour maps of the TRIR spectra and the corresponding fits are shown in Figure S3, and the time-dependent fractional population changes are presented in Figure S4. The temperature- and λ_ex_-dependent time constants for IS decay, monomer formation, and dimer recovery, along with the quantum yield (Φ) for dimer dissociation, are summarized in Table 1.

As summarized in Table 1, the dimer recovery time (τ_r_) from IS increases, whereas the monomer formation time (τ_f_) decreases, as λ_ex_ is tuned from 400 to 350 nm at 293 K. As shown in Figure 1a, both wavelengths excite the same absorption band centered at 375 nm, which is assigned to the S–S bond n → σ* transition. If IS were an ED (Scheme I), neither τ_f_ nor τ_r_ would be expected to depend on λ_ex_, because both wavelengths populate the same excited electronic state and therefore yield similar relaxation and dissociation dynamics. Conversely, if IS is a DR (Scheme II), excitation at different wavelengths generates nascent diradicals with different amounts of excess energy. Excitation at 350 nm therefore produces a more energetic DR than excitation at 400 nm, promoting secondary cleavage of the remaining S–S bond. Consequently, the higher-energy DR undergoes secondary dissociation more rapidly and recombines with lower probability, leading to faster monomer formation, slower dimer recovery, and a higher monomer yield, in excellent agreement with the experimental observations. The temperature dependence under identical excitation conditions (350 nm) provides further support for Scheme II. Increasing the temperature accelerates secondary S–S bond cleavage, resulting in faster monomer formation. At the same time, enhanced thermal motion at higher temperature increases fluctuations within the radical pair, reducing the probability of geminate recombination and thereby slowing dimer recovery. Such temperature dependence is not expected if IS were an ED, because relaxation and dissociation on a single excited-state potential energy surface (PES) should exhibit only modest temperature dependence. Moreover, excited-state lifetimes of many organic molecules are known to decrease with increasing temperature due to enhanced nonradiative relaxation [30,31] in contrast to the present results, where the decay time increases as the temperature is raised from 273 to 293 K. These wavelength- and temperature-dependent behaviors indicate that the species observed during the first few picoseconds is not a simple electronically excited state but a structural intermediate formed immediately after the initial S–S bond cleavage. This intermediate governs the competition between geminate recombination and complete dissociation. As discussed above, the calculated vibrational frequencies of the bis-thiyl diradical obtained using the truncated model compound reproduce the observed IS spectrum well (see Figure 3a). Accordingly, IS is assigned to a bis-thiyl diradical, whose subsequent reaction pathways are governed by its excess energy. Taken together, the spectroscopic signatures, wavelength dependence, temperature dependence, and theoretical calculations consistently support assignment of IS to a bis-thiyl diradical and strongly disfavor the electronically excited dimer model. These results establish Scheme II as the appropriate mechanistic description of the photodissociation dynamics of the CAAC–CS_2_ dimer following excitation of the 375 nm S–S n → σ* transition.

**Figure 5:**
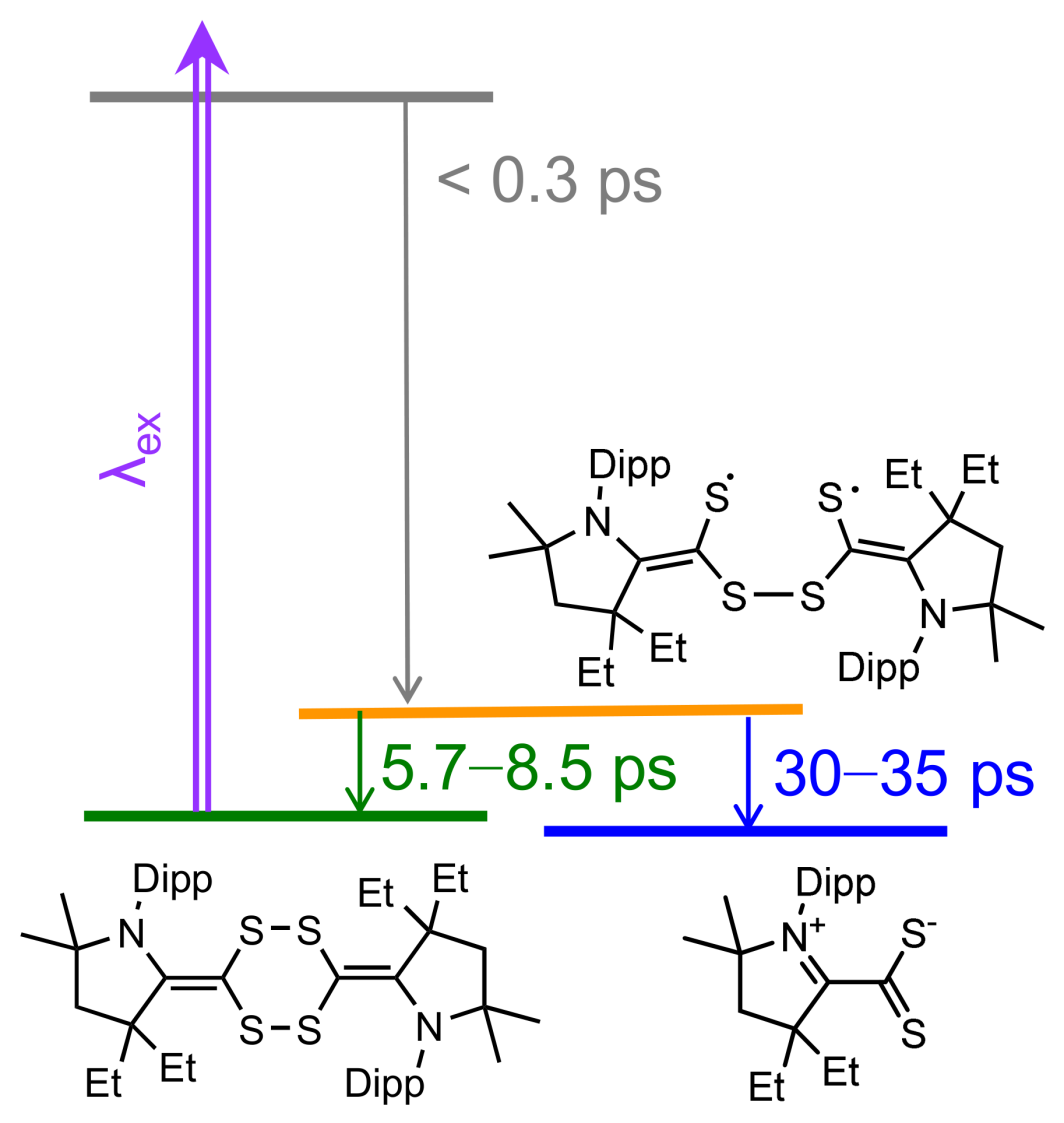
Schematic of the photodissociation dynamics of the CAAC–CS_2_ dimer in CD_2_Cl_2_ at 273 and 293 K following excitation of the 375 nm band. The monomer is thermodynamically more stable than the dimer. Molecular structures of the corresponding states are shown.

Figure 5 schematically illustrates the photodissociation dynamics of the CAAC–CS_2_ dimer in CD_2_Cl_2_ at 273 and 293 K following excitation of the 375 nm absorption band. Immediately after photoexcitation (<0.3 ps), the CAAC–CS_2_ dimer undergoes homolytic cleavage of one of the two S–S bonds, forming a bis-thiyl diradical with excess energy. The diradical can either undergo secondary cleavage of the remaining S–S bond to produce two stable monomers or recombine to regenerate the dimer. This intermediate decays with time constants of 5.0–6.7 ps and partitions between these two competing pathways. As summarized in Table 1, both the decay kinetics of the intermediate and the branching ratio between monomer formation and dimer recovery depend on λ_ex_ and temperature.

**Figure 6:**
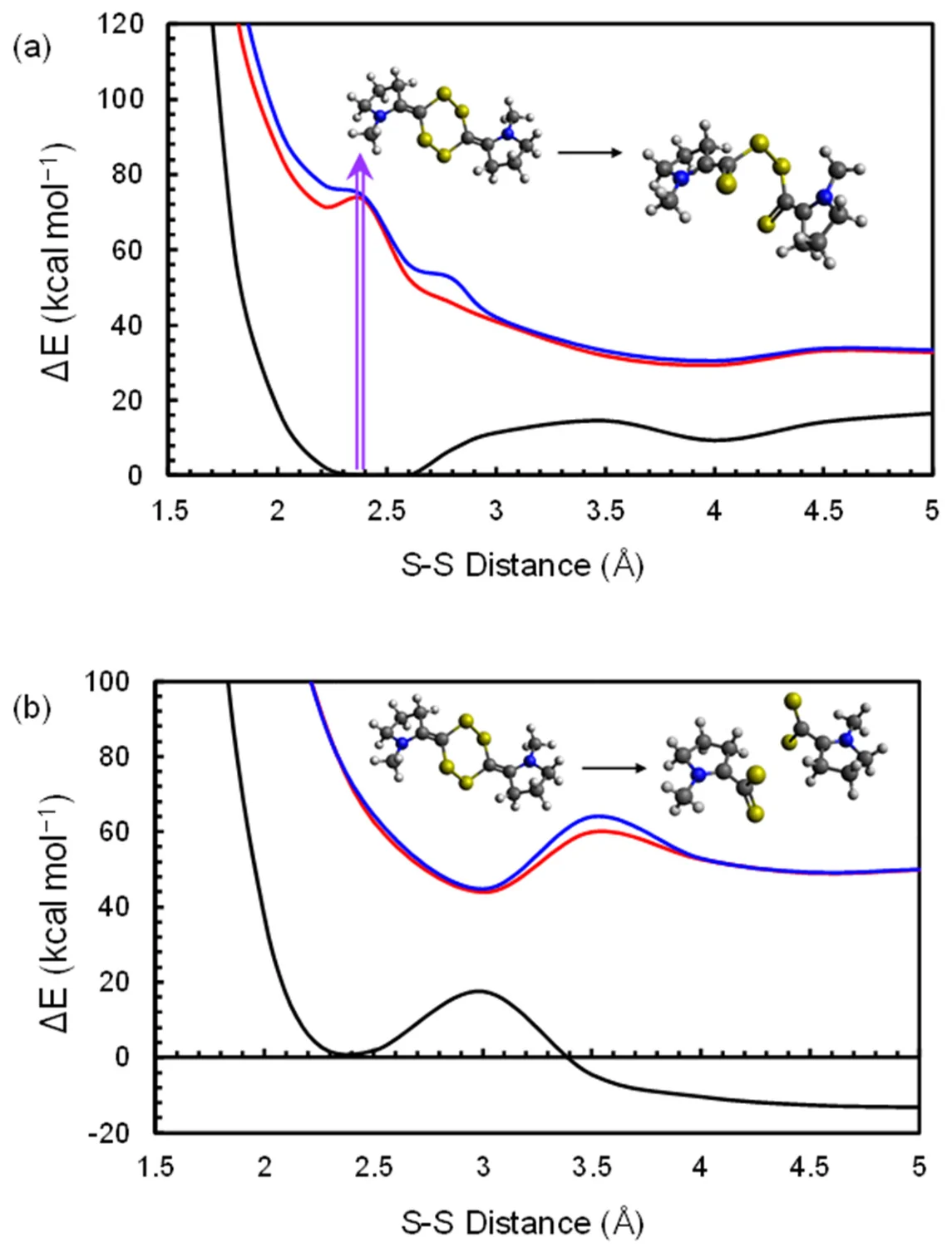
(**a**) Potential energy surfaces (PESs) of the CAAC–CS_2_ dimer as a function of one of the two S–S bond distances, calculated at the XMS-CASPT2/6-31G level. The S_1_ (red) and S_2_ (blue) states arise from S n → S–S σ* excitations and lead to direct homolytic cleavage of a single S–S bond. The purple arrow denotes the excitation energy. (**b**) PESs of the CAAC–CS_2_ dimer as a function of both S–S bond distances, calculated at the same level. Although the S_1_ (red) and S_2_ (blue) states also correspond to S n → S–S σ* excitations, the excited-state surfaces are not fully dissociative along the concerted coordinate, disfavoring simultaneous cleavage of both S–S bonds. The ground-state surface (black) confirms that the monomer is more stable than the dimer. Because multireference calculations on the full CAAC–CS_2_ dimer are computationally demanding, a truncated model compound was employed. The structures of the model compound and relevant intermediates are shown.

To elucidate the mechanism of S–S bond dissociation, multireference electronic structure calculations were performed. The PESs of the CAAC–CS_2_ dimer were explored as a function of one of the two S–S bond distances using CASSCF calculations. Dynamic electron correlation was included using extended multistate CAS second-order perturbation theory [32] (XMS-CASPT2) with the 6-31G basis set [33]. Because direct multireference calculations on the full CAAC–CS_2_ dimer are computationally demanding, a truncated model compound was employed for PES analysis (Figure 6). The calculated PESs obtained by elongating one of the two S–S bonds indicate that the S_1_ and S_2_ excited-state surfaces are dissociative along this coordinate (Figure 6a). This finding is consistent with the experimental observation that one S–S bond cleaves within 0.3 ps after photoexcitation and supports prompt formation of a diradical upon excitation. To evaluate the possibility of concerted cleavage, PESs were also calculated as a function of both S–S bond distances at the XMS-CASPT2/6-31G level (Figure 6b). The ground-state surface confirms that the monomer is more stable than the dimer, whereas the excited-state surfaces exhibit barriers along the concerted dissociation pathway in both the S_1_ and S_2_ states. These features disfavor concerted cleavage of both S–S bonds. Overall, the PES analysis qualitatively supports a stepwise dissociation mechanism involving sequential S–S bond cleavage rather than a concerted process. We attempted TD-DFT calculations for the full molecule but found that the calculations did not converge. Indeed, adiabatic TD-DFT has been reported to fail qualitatively for excited-state PESs involving homolytic bond dissociation [34,35].

A previous theoretical study, based on ground-state DFT calculations of a truncated model compound, proposed a stepwise dissociation mechanism involving a bis-thiyl diradical intermediate [16]. DR was calculated to lie 13.4 kcal/mol above the dimer, and the barrier for the secondary S–S cleavage (5.7 kcal/mol) was 1 kcal/mol higher than that for recombination to the dimer. Experimentally, the barrier difference estimated from the observed rates of secondary dissociation and recombination of IS is ~0.8 kcal/mol, with secondary dissociation being higher in energy. This agreement supports assignment of IS as a bis-thiyl DR.

To further assess the concerted dissociation pathway, a solvent-caged CAAC–CS_2_ GP with incomplete separation was modeled by constraining the separation between the two fragments following cleavage of both S–S bonds, and its vibrational frequencies were computed. Although the calculated spectrum exhibits a red-shifted C=C stretching mode consistent with the IS spectrum, the predicted extinction coefficient is larger than that of the dimer, contrary to the experimental observation. In the experimentally determined structure, the CAAC–CS_2_ dimer adopts an approximately planar geometry with an N–C–C–S torsion angle of 11.7°, whereas the monomer is markedly nonplanar with a torsion angle of 91.7° [16]. Following concerted cleavage of both S–S bonds, a strong driving force exists for rotation about the N–C–C–S torsion, converting the C=C character into C=N character. The PES of CAAC–CS_2_ as a function of this torsion angle, calculated at the B3LYP-D3/def2-SVP level (Figure S5), indicates that this rotation is energetically favorable. Consequently, the initially formed GP should relax rapidly, making it short-lived and unlikely to accumulate to a detectable population, even within the solvent cage. Thus, CAAC–CS_2_ is expected to form essentially immediately after concerted dissociation of the CAAC–CS_2_ dimer, and no distinct intermediate spectrum would be expected in the TRIR data. On this basis, the concerted dissociation mechanism can be ruled out.

## 3. Materials and Methods

### 3.1. Sample Preparation

The CAAC–CS_2_ dimer and monomer were synthesized according to previously reported procedures [16]. CD_2_Cl_2_ (Sigma-Aldrich, St. Louis, MO, USA) was used as received. The CAAC–CS_2_ dimer (3.5–5 mM) was dissolved in CD_2_Cl_2_ that had been thoroughly degassed by bubbling with N_2_ to remove dissolved O_2_. All solutions were prepared under a nitrogen atmosphere to minimize exposure to moisture and oxygen. The equilibrated solutions were loaded into a gas-tight sample cell (100 μm path length) equipped with two 2 mm-thick CaF_2_ windows. Concentration and path length were optimized to provide sufficient transient absorption signals while avoiding excessive optical density. UV–vis and FT-IR spectra were recorded throughout the experiments to ensure sample integrity. The sample temperature was maintained at 293 ± 1 or 273 ± 1 K by circulating coolant through an aluminum block in contact with the cell.

### 3.2. TRIR Spectroscopy

The TRIR spectrometer has been described previously [36,37]. Briefly, a commercial Ti:sapphire oscillator/amplifier system (Spitfire Ace, Spectra Physics (Milpitas, CA, USA)) produced 100 fs pulses at 800 nm with a pulse energy of 700 μJ at a 2 kHz repetition rate. The 800 nm output was split and sent to two custom-made optical parametric amplifiers (OPAs) to generate near-IR signal and idler pulses. The near-IR output from one OPA was difference-frequency mixed in a 1 mm-thick type I AgGaS_2_ crystal to generate tunable mid-IR probe pulses (120 fs, 160 cm^−1^ bandwidth). The samples were excited with either 400 or 350 nm pump pulses. The 400 nm pulses (140 fs, 10 μJ) were generated by second-harmonic generation (SHG) of the 800 nm fundamental in a 0.6 mm type I BBO crystal. The 350 nm pulses (140 fs, 3 μJ) were generated by frequency doubling the signal output of the second OPA in a 0.6 mm type I BBO crystal, followed by a second SHG step in a 0.5-mm type I BBO crystal. The pump–probe delay was controlled using a mechanical delay stage. The pump polarization was set to the magic angle (54.7°) relative to the probe to ensure isotropic detection and minimize polarization artifacts. To suppress nonlinear effects, the pump pulse energy at the sample was attenuated to 0.5 μJ. The mid-IR probe pulse (10 nJ) was transmitted through the pump-excited sample. Spectral coverage beyond the intrinsic probe bandwidth (160 cm^−1^) was obtained by combining overlapping spectra acquired at probe center frequencies of 1520 and 1480 cm^−1^. The transmitted probe was dispersed with a 320 mm monochromator (HR320, Horiba, Miami, FL, USA) equipped with a 100 lines/mm grating and detected using a liquid-nitrogen-cooled 1 × 128 pixel HgCdTe array detector (MCT-8-129, Infrared Associates, Stuart, FL, USA), providing a spectral resolution of 1.5–1.6 cm^−1^ pixel^−1^. The detector output was amplified and digitized with 16-bit A/D converters. The pump beam was modulated at 1 kHz using an optical chopper (MC1000A, Thorlabs, Newton, NJ, USA) synchronized to the 2 kHz laser repetition rate, enabling shot-to-shot differential detection between excited and unexcited signals. The instrument response function, determined from the pump–probe cross-correlation in a Si wafer, was approximately 0.2 ps.

### 3.3. Computational Details

The ground-state equilibrium structures of the CAAC–CS_2_ dimer and CAAC–CS_2_ were optimized using DFT with the B3LYP-GD3 functional and the def2-SVP basis set [26,27]. Solvent effects were included with the polarizable continuum model. Harmonic vibrational frequencies were computed for the optimized structures and scaled for comparison with the experimental spectra. Multireference calculations were performed to probe the excited-state electronic structure of the CAAC–CS_2_ dimer. CASSCF calculations employed an active space of 12 electrons in 10 orbitals, encompassing the S n, C=C π, S–S σ*, and C=C π* orbitals relevant to dissociation. All calculations were performed using the OpenMOLCAS software package version 26.06 [38,39,40]. Dynamic electron correlation was included using XMS-CASPT2 version 26.06 [28,41,42]. A state-averaged Fock operator over three states (S_0_–S_2_) was used to ensure a balanced description. An ionization potential–electron affinity (IPEA) shift of 0.25 a.u. was applied, and both real and imaginary shifts were set to 0.00 a.u. to avoid intruder states [43,44]. PESs were computed as a function of one or both S–S bond distances to elucidate dissociation pathways (Figure 6). The PESs indicate that S–S bond cleavage proceeds readily upon excitation to the dissociative S_1_ state. Consistent with this assignment, the calculated molecular orbitals (Figure S6) show that the S_1_ and S_2_ states are dominated by S–S n → σ* transitions.

## 4. Conclusions

In summary, the photodissociation dynamics of the CAAC–CS_2_ dimer in CD_2_Cl_2_ at 293 and 273 K were investigated by femtosecond TRIR spectroscopy following excitation at 400 and 350 nm, complemented by electronic structure calculations. Photoexcitation triggers ultrafast homolytic cleavage of one S–S bond to form a bis-thiyl diradical, which evolves on the picosecond timescale via two competing pathways: recombination to regenerate the dimer and secondary S–S bond cleavage to yield two stable monomers. The relative rates and branching between these pathways depend on both excitation wavelength and temperature. Higher-energy excitation (350 nm) increases the excess energy in the nascent fragments, thereby suppressing recombination and promoting monomer formation via faster secondary dissociation. Conversely, lowering the temperature to 273 K slows secondary dissociation and reduces thermal fluctuations within the radical pair, favoring recombination. Together, these results provide direct spectroscopic insight into ultrafast dual S–S bond cleavage and the associated reaction dynamics, highlighting the critical roles of excess energy and radical-pair thermal fluctuations in determining intermediate fate in photoexcited sulfur–sulfur-bonded systems.

## Figures and Tables

**Table 1:** Temperature- and excitation wavelength (λ_ex_)-dependent time constants for the species involved in the photodissociation dynamics of the CAAC–CS_2_ dimer in CD_2_Cl_2_. The quantum yields (Φ) for dimer dissociation are also listed. The time constants for decay of the intermediate species (IS), formation of the monomer, and recovery of the dimer are denoted τ_d_, τ_f_, and τ_r_, respectively.

Temp. (K)	λ_ex_ (nm)	τ_d_ (ps)	τ_f_ (ps)	τ_r_ (ps)	Φ
293	400	5.0	35	5.7	0.14
293	350	6.7	30	8.5	0.22
273	350	6.0	35	7.2	0.17

## Data Availability

The original contributions presented in this study are included in the article and Supplementary Materials. Further inquiries can be directed to the corresponding authors.

## Supplementary Materials

The following supporting information can be downloaded at: https://www.mdpi.com/article/10.3390/ijms27167190/s1.

## Abbreviations

The following abbreviations are used in this manuscript:

CAAC	Cyclic(alkyl)(amino)carbene
TRIR	Time-Resolved infrared
FTIR	Fourier transform infrared
UV-vis	Ultraviolet–visible
TD-DFT	Time-dependent density functional thoery
DFT	Density functional theory
CASSCF	Complete active space self-consistent field
XNS-CASPT2	Extended multistate complete active space second-order perturbation theory
DR	Diradical
IS	Intermediate species
ED	Excited dimer
GP	Geminate pair
PES	Potential energy surface
OPA	Optical parametric amplifier
MO	Molecular orbital
SHG	Second-harmonic generation
