# Supplementary material for: Ultrafast Photochemical Reaction Dynamics of a Cyclic (Alkyl)(Amino)Carbene-Carbon Disulfide Dimer Probed by Femtosecond Infrared Spectroscopy
Source: Int J Mol Sci. 2026 Aug 11;27(16):7190. doi: 10.3390/ijms27167190 (PMC13513443; doi:10.3390/ijms27167190)
Supplement: Supplementary file 1 [file ijms-27-07190-s001.zip › ijms-4471649-supplementary.pdf]

Supporting Information

Ultrafast Photochemical Reaction Dynamics of a Cyclic (Alkyl)(Amino)Carbene-Carbon Disulfide Dimer Probed by Femtosecond Infrared Spectroscopy

Seongbeom Jeon[†1,2], *Juhyang Shin*[†1,2,3], *Jaegeum Cha*[1,3], *Youngsuk Kim*[*1,2,3] *and Manho Lim*[*1,2]

[1] Department of Chemistry, Pusan National University, Busan 46241, Korea

[2] Chemistry Institute for Functional Materials, Pusan National University, Busan 46241, Korea

[3] Institute for Future Earth, Pusan National University, Busan 46241, Korea

[†]These authors contributed equally

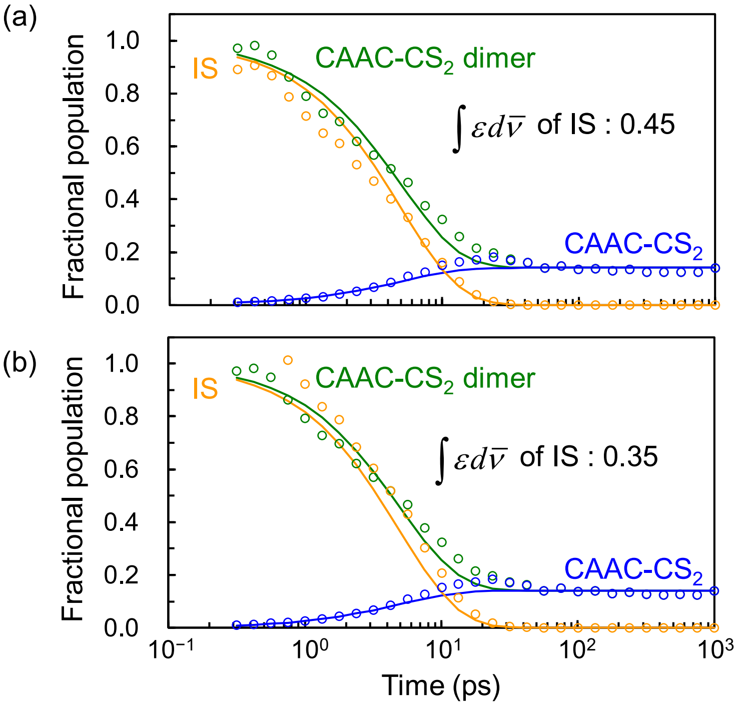

Figure S1. Fractional population changes obtained when the integrated extinction coefficient ($\int \varepsilon d\bar{\nu}$) of IS is assumed to be (a) 0.45 or (b) 0.35 times that of the dimer. As shown in the figure, these values result in a much poorer fit to the data, as indicated by the discrepancy between the orange circles (data) and orange lines (fits).

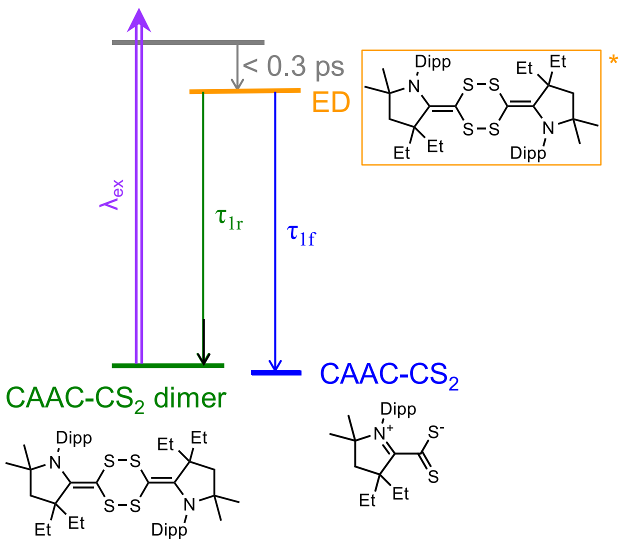

Scheme 1

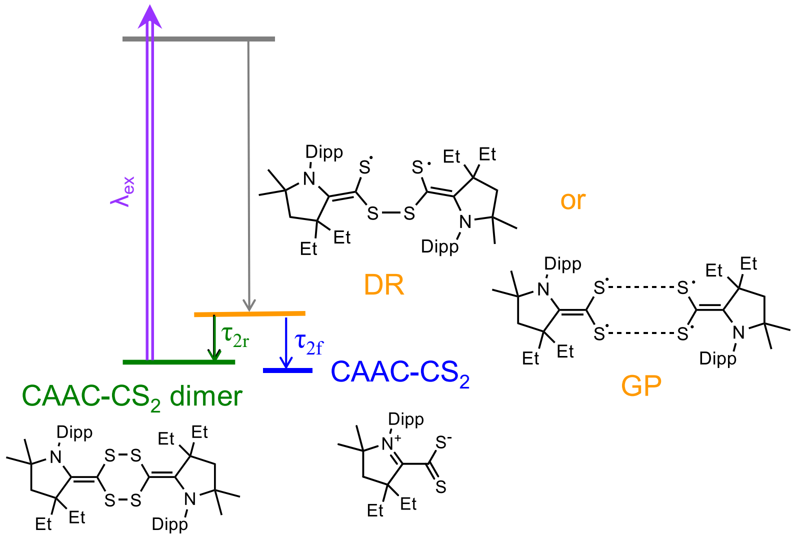

Scheme 2

Figure S2. Schematics of the possible time-dependent populations of species formed upon photoexcitation of the CAAC–CS_2 dimer in CD_2Cl_2 at 293 K (λ_{exc} = 400 nm). (Scheme I) The intermediate state (IS) is assigned to the electronically excited CAAC–CS_2 dimer (ED). ED either relaxes to the ground-state dimer with a time constant τ_{1r} or dissociates into monomers with a time constant τ_{1f}. (Scheme II) IS is assigned either to a bis-thiyl diradical (DR) or to a dissociated nascent product that remains partially separated within the solvent cage (a geminate pair; GP). DR either recombines to regenerate the dimer with a time constant τ_{2r} or undergoes secondary cleavage of the remaining S–S bond to yield two stable monomers with a time constant τ_{2f}. GP either recombines geminately to form the dimer with a time constant τ_{2r} or escapes the solvent cage to form stable monomers with a time constant τ_{2f}. Molecular structures of the corresponding states are shown. The molecular structure of ED is represented by an asterisk (*) in that of the ground state.

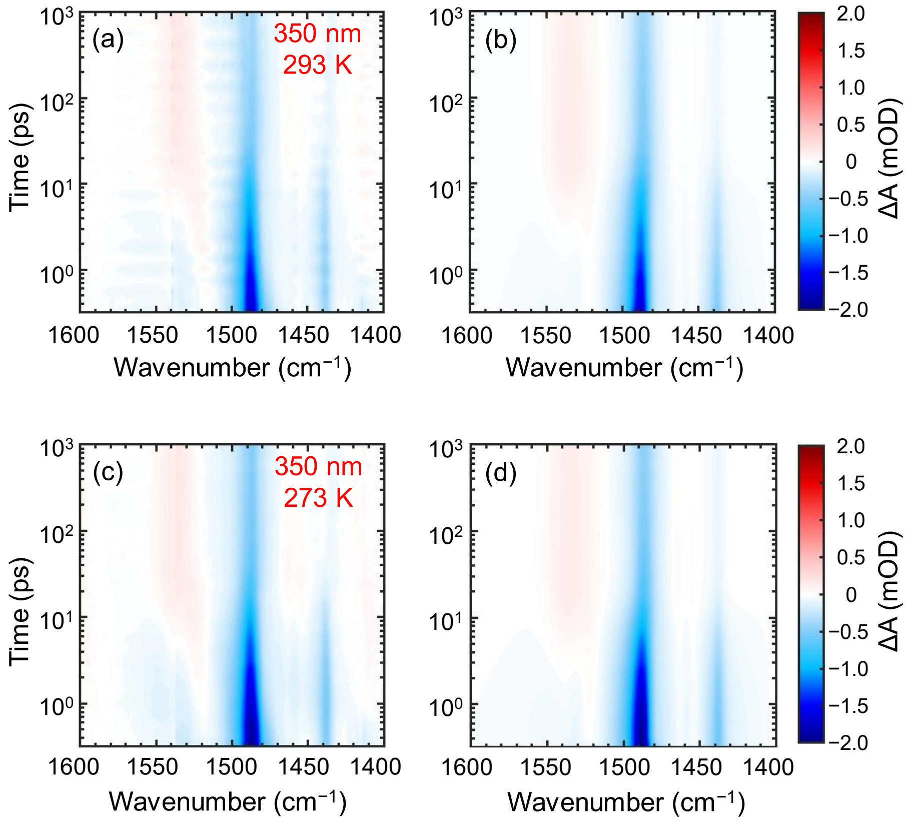

Figure S3. Two-dimensional contour maps of femtosecond TRIR spectra of the CAAC–CS_2 dimer in CD_2Cl_2: (a) at 293 K following excitation at 350 nm and (b) at 273 K following excitation at 350 nm. Panels (c) and (d) show the corresponding fits at 293 and 273 K, respectively.

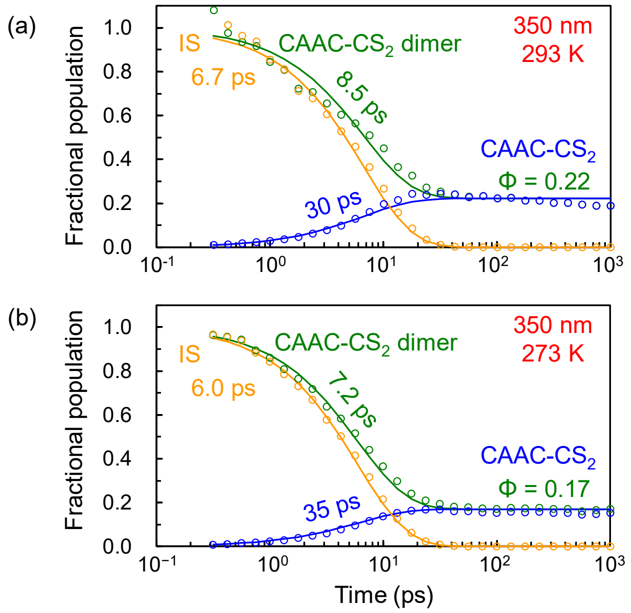

Figure S4. Time-dependent changes in the fractional populations of species associated with the three basis spectra involved in the photodissociation of the CAAC–CS_2 dimer: (a) at 293 K following excitation at 350 nm and (b) at 273 K following excitation at 350 nm. The CAAC-

CS_2 dimer signal reflects depletion of the ground-state dimer, whereas the IS and CAAC–CS_2 signals report the populations of the intermediate species (IS) and CAAC–CS_2, respectively. Time constants obtained from exponential fits to the kinetics, together with the quantum yield (Φ) for dimer dissociation, are indicated.

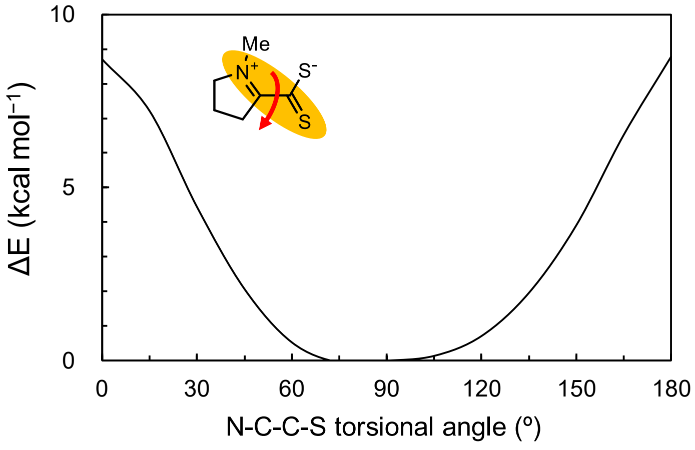

Figure S5. Potential energy surfaces (PESs) of CAAC–CS_2 as a function of the N−C−C−S dihedral angle, calculated at the XMS-CASPT2/6-31G level.

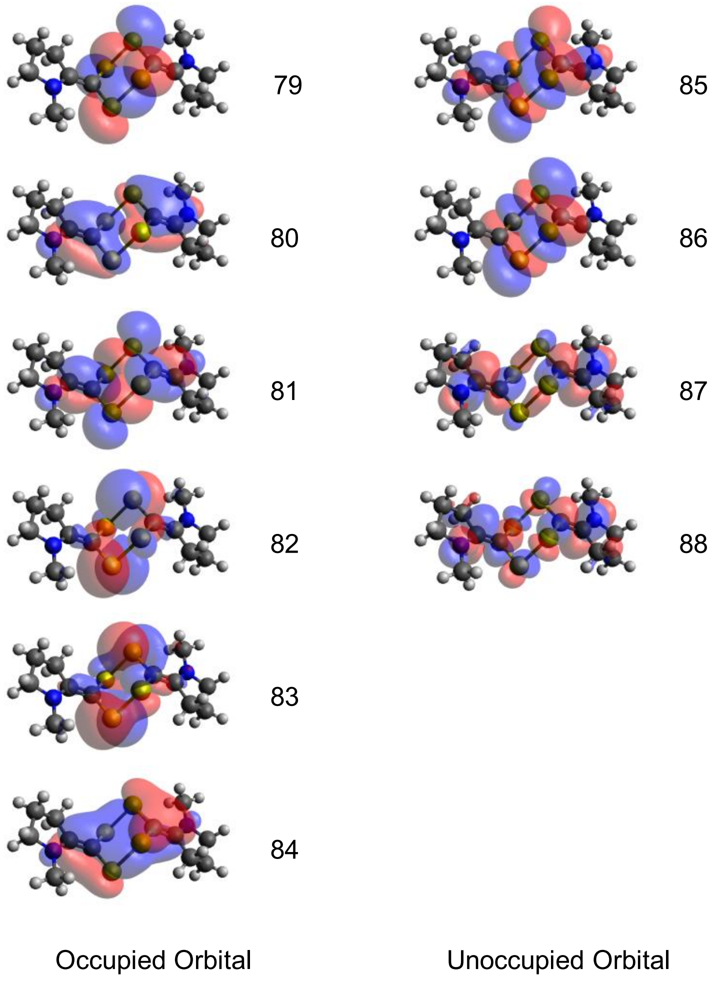

Figure S6. Representative occupied and unoccupied molecular orbitals (MOs) of the CAAC-CS_2 dimer, including the HOMO and LUMO, calculated at the CASSCF(12,10)/6-31G level.

Table S1. Harmonic IR frequencies of CAAC–CS_2 dimer and monomer at the B3LYP-GD3/def2-SVP level. Vibrational frequencies were scaled by factors of 0.980 and 0.969 for the CAAC–CS_2 dimer and monomer, respectively.

	CAAC–CS_2 dimer		CAAC–CS_2	
Vibration mode	Frequency (cm^{-1})	Intensity (km/mol)	Frequency (cm^{-1})	Intensity (km/mol)
ν_1	10.33	0.0766	23.77	0.7820
ν_2	16.13	1.0652	38.97	0.3440
ν_3	27.22	0.0000	40.21	0.0472
ν_4	30.15	0.1843	75.01	0.9108
ν_5	33.35	1.2199	85.32	0.5855
ν_6	38.70	0.0000	105.22	4.8574
ν_7	47.14	0.0000	114.69	0.0039
ν_8	52.68	0.2968	133.06	3.5575
ν_9	54.18	0.0000	139.32	0.8948
ν_{10}	67.88	0.1517	150.89	0.7014
ν_{11}	71.01	0.0000	154.29	1.1523
ν_{12}	76.31	0.0000	162.29	0.4842
ν_{13}	88.46	1.0556	168.75	0.0601
ν_{14}	99.85	0.0000	172.66	1.1176
ν_{15}	105.42	0.2739	174.99	0.1627
ν_{16}	108.27	0.0000	184.46	0.5087
ν_{17}	108.41	0.4900	185.25	0.2789
ν_{18}	111.37	0.0000	192.88	0.7291
ν_{19}	118.30	0.1805	199.81	0.1608
ν_{20}	124.22	0.2528	212.97	3.6523
ν_{21}	125.74	0.0000	228.05	0.0136
ν_{22}	133.03	0.0000	240.90	3.0402
ν_{23}	138.50	0.0000	250.67	3.6706
ν_{24}	140.03	0.2348	256.36	4.6002
ν_{25}	143.21	0.3590	259.79	0.1526
ν_{26}	144.69	0.0000	279.60	2.0306
ν_{27}	146.07	0.1850	283.18	0.2926
ν_{28}	151.96	0.0000	289.91	0.2158
ν_{29}	155.74	0.0000	294.50	0.9393
ν_{30}	156.60	2.0160	298.84	1.2753
ν_{31}	164.73	0.9211	315.44	0.0172
ν_{32}	175.88	0.0000	325.76	0.0914
ν_{33}	192.62	3.2081	350.94	0.5423
ν_{34}	196.15	3.7774	362.17	0.2628
ν_{35}	201.85	0.0000	380.64	0.2402
ν_{36}	209.40	0.0001	389.80	1.8885
ν_{37}	209.60	0.9000	402.68	1.1645
ν_{38}	209.90	1.9705	422.18	7.1394
ν_{39}	210.12	0.0001	433.98	0.0113

ν_{40}	212.11	0.0000	452.25	0.4629
ν_{41}	218.60	2.1246	462.46	0.0615
ν_{42}	227.97	0.0000	468.23	0.0592
ν_{43}	230.77	1.2171	482.86	6.6082
ν_{44}	232.37	0.0000	492.01	0.6246
ν_{45}	237.36	0.0000	538.25	4.3548
ν_{46}	240.37	4.3628	551.87	5.8381
ν_{47}	241.45	0.0000	565.39	0.1783
ν_{48}	245.88	7.8584	594.54	5.2335
ν_{49}	248.86	0.0000	602.24	12.3158
ν_{50}	249.36	0.3261	607.28	0.0198
ν_{51}	255.91	0.0000	634.10	2.7427
ν_{52}	264.82	3.0978	696.79	8.5058
ν_{53}	267.16	4.3197	704.93	6.6842
ν_{54}	267.83	0.0000	725.08	10.7630
ν_{55}	272.35	0.0000	757.83	6.1481
ν_{56}	274.81	1.0096	763.15	0.9539
ν_{57}	280.61	0.0000	780.18	27.8347
ν_{58}	284.42	1.5082	798.21	1.7768
ν_{59}	290.22	0.0000	800.07	12.6595
ν_{60}	291.57	0.6152	807.48	29.0870
ν_{61}	299.61	0.0000	833.07	11.1140
ν_{62}	300.40	0.1627	858.20	4.3842
ν_{63}	304.25	0.0000	873.62	0.9574
ν_{64}	305.45	0.2812	910.75	6.7931
ν_{65}	318.20	0.0000	912.28	0.2706
ν_{66}	319.42	0.3133	913.77	4.4018
ν_{67}	322.36	2.3064	916.71	18.7380
ν_{68}	323.14	0.0000	918.78	12.0147
ν_{69}	325.71	0.0000	920.92	14.9637
ν_{70}	327.77	0.6088	931.39	0.2405
ν_{71}	345.88	13.0841	933.15	0.4660
ν_{72}	351.46	0.0000	943.85	10.0682
ν_{73}	358.28	0.0000	949.04	1.6078
ν_{74}	364.33	6.5459	966.16	26.3649
ν_{75}	372.40	46.9202	987.99	4.0216
ν_{76}	375.57	0.0001	993.20	1.5176
ν_{77}	381.72	12.2059	1000.36	7.9900
ν_{78}	382.20	0.0000	1014.10	18.8448
ν_{79}	401.09	3.5231	1028.62	385.9977
ν_{80}	409.43	0.0000	1034.84	13.9184
ν_{81}	411.02	0.0000	1044.58	51.0938
ν_{82}	414.63	5.2930	1054.51	1.8670
ν_{83}	430.66	10.7904	1055.35	3.1415
ν_{84}	432.36	0.0000	1062.39	14.6918
ν_{85}	449.15	16.5160	1073.21	2.6153
ν_{86}	451.34	0.0000	1075.27	0.8683
ν_{87}	458.25	0.0000	1077.68	26.5103
ν_{88}	459.03	0.5389	1109.43	32.8061

ν_{89}	474.43	0.0004	1132.41	23.5466
ν_{90}	474.77	118.0781	1139.52	12.3768
ν_{91}	490.96	0.0000	1144.45	3.7512
ν_{92}	492.25	3.6482	1146.18	23.1759
ν_{93}	501.89	0.0000	1155.28	20.4532
ν_{94}	503.14	55.1429	1156.58	38.7544
ν_{95}	536.22	5.8990	1164.48	6.8203
ν_{96}	536.24	0.0000	1206.42	1.4759
ν_{97}	558.10	4.6887	1225.21	1.0102
ν_{98}	558.11	0.0018	1235.58	3.6530
ν_{99}	562.57	0.0000	1247.86	20.3597
ν_{100}	562.95	5.8863	1251.30	0.3372
ν_{101}	600.21	30.1789	1261.42	2.3400
ν_{102}	600.56	0.0000	1262.91	7.2396
ν_{103}	604.64	8.6258	1271.06	1.5500
ν_{104}	604.80	0.0000	1272.17	0.4435
ν_{105}	614.19	5.9502	1298.66	3.8519
ν_{106}	614.40	0.0000	1305.77	2.3288
ν_{107}	643.20	2.9934	1307.34	22.1288
ν_{108}	643.21	0.0006	1320.08	3.4960
ν_{109}	675.24	107.9408	1330.92	9.5666
ν_{110}	678.83	0.0000	1342.85	11.0628
ν_{111}	687.39	18.4373	1343.96	1.4541
ν_{112}	689.18	0.0000	1344.48	3.7908
ν_{113}	730.40	21.8473	1353.18	2.6947
ν_{114}	731.00	0.0000	1356.56	41.3912
ν_{115}	762.62	8.3527	1364.05	11.6424
ν_{116}	763.87	0.0000	1365.39	3.5609
ν_{117}	773.82	0.0014	1365.60	4.3528
ν_{118}	773.86	39.9777	1370.84	7.9821
ν_{119}	785.82	19.6397	1401.40	0.8089
ν_{120}	787.32	0.0000	1406.96	3.9739
ν_{121}	789.16	46.8224	1408.64	1.7971
ν_{122}	791.04	0.0000	1410.24	2.7506
ν_{123}	804.46	12.1824	1411.64	7.8310
ν_{124}	804.65	0.0000	1413.50	0.9205
ν_{125}	818.10	34.7177	1413.95	2.6653
ν_{126}	818.44	0.0001	1416.02	11.9974
ν_{127}	835.67	11.2674	1417.33	0.0502
ν_{128}	837.11	0.0000	1418.96	1.7561
ν_{129}	850.02	42.1753	1422.68	3.6774
ν_{130}	856.29	0.0000	1424.79	0.0079
ν_{131}	880.82	4.4146	1429.20	5.9551
ν_{132}	880.91	0.0000	1429.75	15.2999
ν_{133}	895.83	31.4655	1431.77	2.0572
ν_{134}	896.70	0.0000	1434.00	17.1148
ν_{135}	918.56	0.2216	1438.36	64.1132
ν_{136}	918.59	0.0000	1439.96	0.5829
ν_{137}	919.89	0.0182	1443.84	24.2521

ν_{138}	919.89	6.9803	1453.37	6.1880
ν_{139}	921.69	0.0006	1460.49	4.2046
ν_{140}	921.72	2.8343	1533.39	433.4345
ν_{141}	922.39	10.2506	1586.06	7.7803
ν_{142}	922.40	0.0006	1593.07	23.2550
ν_{143}	930.55	31.7098	2936.80	28.8561
ν_{144}	930.60	0.0005	2937.05	27.3447
ν_{145}	938.91	12.7711	2942.33	42.0232
ν_{146}	938.96	0.0000	2944.05	14.7950
ν_{147}	941.51	0.0000	2945.04	65.7441
ν_{148}	941.67	3.3739	2945.34	27.1425
ν_{149}	945.72	0.0672	2945.37	12.2449
ν_{150}	945.74	0.0002	2951.11	72.6341
ν_{151}	946.40	0.0012	2959.01	14.5343
ν_{152}	946.41	1.3089	2964.42	16.8404
ν_{153}	959.47	0.0001	3000.04	7.8122
ν_{154}	959.51	4.4287	3000.99	16.1841
ν_{155}	989.66	7.4776	3003.92	14.5478
ν_{156}	989.70	0.0002	3004.03	20.2867
ν_{157}	996.84	0.9436	3006.83	5.2143
ν_{158}	996.85	0.0004	3008.71	0.6256
ν_{159}	1004.09	3.9888	3009.11	0.7245
ν_{160}	1004.16	0.0000	3015.89	19.7088
ν_{161}	1023.29	0.0000	3018.22	80.8793
ν_{162}	1023.37	0.6122	3023.29	24.8225
ν_{163}	1028.47	14.4147	3024.04	107.4389
ν_{164}	1028.58	0.0000	3038.15	7.8364
ν_{165}	1039.48	5.3188	3039.62	10.1102
ν_{166}	1040.22	0.0000	3040.82	24.3422
ν_{167}	1044.90	25.8639	3044.90	38.6836
ν_{168}	1045.22	0.0000	3046.03	8.4341
ν_{169}	1053.66	0.0108	3050.43	25.2561
ν_{170}	1053.68	38.3836	3053.27	17.9993
ν_{171}	1058.75	3.5304	3057.79	12.7326
ν_{172}	1058.88	0.0000	3058.54	21.9406
ν_{173}	1080.00	16.5339	3070.81	13.7024
ν_{174}	1080.14	0.0000	3073.68	14.0407
ν_{175}	1096.26	12.4138	3082.86	3.6798
ν_{176}	1096.29	0.0017	3098.46	13.9658
ν_{177}	1099.34	4.5181	3105.56	18.7480
ν_{178}	1099.35	0.0002		
ν_{179}	1105.41	24.6067		
ν_{180}	1105.48	0.0009		
ν_{181}	1137.87	121.7510		
ν_{182}	1137.96	0.0001		
ν_{183}	1146.67	0.0000		
ν_{184}	1146.70	1.3159		
ν_{185}	1157.78	163.6018		
ν_{186}	1158.55	0.0000		

ν_{187}	1159.18	17.1167
ν_{188}	1159.28	0.0001
ν_{189}	1172.00	128.2031
ν_{190}	1172.44	0.0001
ν_{191}	1173.93	13.6914
ν_{192}	1173.93	4.9580
ν_{193}	1183.78	23.8947
ν_{194}	1184.07	0.0000
ν_{195}	1190.27	335.2574
ν_{196}	1191.84	0.0000
ν_{197}	1217.94	110.0073
ν_{198}	1218.69	0.0000
ν_{199}	1240.47	12.0595
ν_{200}	1240.57	0.0000
ν_{201}	1248.28	4.2753
ν_{202}	1248.28	16.9541
ν_{203}	1263.43	0.0001
ν_{204}	1263.48	13.4054
ν_{205}	1269.56	17.7382
ν_{206}	1269.67	0.0001
ν_{207}	1270.91	0.0004
ν_{208}	1270.92	2.0441
ν_{209}	1300.91	25.6904
ν_{210}	1301.01	0.0064
ν_{211}	1302.44	40.6279
ν_{212}	1303.07	0.0000
ν_{213}	1311.04	0.0289
ν_{214}	1311.12	12.8693
ν_{215}	1317.84	9.9378
ν_{216}	1317.88	0.0453
ν_{217}	1326.95	82.1264
ν_{218}	1327.41	0.0000
ν_{219}	1330.64	9.3598
ν_{221}	1330.83	0.0000
ν_{222}	1336.76	47.8353
ν_{223}	1336.83	0.0048
ν_{224}	1348.77	743.1450
ν_{225}	1351.04	0.0001
ν_{226}	1351.66	28.6986
ν_{227}	1352.14	0.0000
ν_{228}	1354.84	17.3080
ν_{229}	1355.09	0.0000
ν_{230}	1360.74	2.9930
ν_{231}	1360.99	0.0000
ν_{232}	1364.12	47.7525
ν_{233}	1364.23	0.0002
ν_{234}	1371.48	9.5234
ν_{235}	1371.49	0.0016
ν_{236}	1374.70	0.0001

ν_{237}	1374.74	12.7549
ν_{238}	1378.36	9.7041
ν_{239}	1378.36	0.1859
ν_{240}	1378.93	14.6238
ν_{241}	1379.30	0.0000
ν_{242}	1380.96	42.7883
ν_{243}	1381.01	0.0006
ν_{244}	1422.50	0.0000
ν_{245}	1422.66	0.3279
ν_{246}	1423.52	12.1215
ν_{247}	1423.54	0.0000
ν_{248}	1425.41	3.2508
ν_{249}	1425.62	0.0000
ν_{250}	1426.71	0.0002
ν_{251}	1426.77	6.0219
ν_{252}	1428.99	40.8785
ν_{253}	1429.16	0.0001
ν_{254}	1429.77	0.0000
ν_{255}	1429.95	4.0226
ν_{256}	1430.60	0.0000
ν_{257}	1430.75	6.8629
ν_{258}	1434.14	0.0000
ν_{259}	1434.33	12.2278
ν_{260}	1435.27	10.2654
ν_{261}	1435.31	0.0014
ν_{262}	1437.29	4.6571
ν_{263}	1437.32	0.0001
ν_{264}	1439.50	7.2607
ν_{265}	1439.68	0.0000
ν_{266}	1442.34	0.0000
ν_{267}	1442.51	35.4849
ν_{268}	1442.67	12.7921
ν_{269}	1442.75	0.0001
ν_{270}	1443.95	68.8802
ν_{271}	1444.08	0.0002
ν_{272}	1446.53	0.0000
ν_{273}	1446.67	16.5534
ν_{274}	1449.51	53.2884
ν_{275}	1449.80	0.0000
ν_{276}	1450.71	7.6398
ν_{277}	1451.10	0.0000
ν_{278}	1453.24	0.0011
ν_{279}	1453.30	25.3226
ν_{280}	1455.05	133.0173
ν_{281}	1455.07	0.0279
ν_{282}	1468.27	38.3699
ν_{283}	1468.57	0.0000
ν_{284}	1470.30	0.0053
ν_{285}	1470.31	31.1790

ν_{286}	1484.66	2943.3092
ν_{287}	1507.22	0.0000
ν_{288}	1599.42	14.6820
ν_{289}	1599.46	0.0000
ν_{290}	1610.95	0.0030
ν_{291}	1610.95	4.2774
ν_{292}	2965.39	44.2340
ν_{293}	2965.39	2.8683
ν_{294}	2965.72	74.4244
ν_{295}	2965.78	0.0000
ν_{296}	2968.64	31.3628
ν_{297}	2968.64	0.0035
ν_{298}	2969.61	57.1709
ν_{299}	2969.62	0.0065
ν_{300}	2971.56	147.8972
ν_{301}	2971.57	0.0095
ν_{302}	2972.26	113.1923
ν_{303}	2972.33	0.0010
ν_{304}	2980.21	137.4524
ν_{305}	2980.31	0.0010
ν_{306}	2986.45	31.8084
ν_{307}	2986.45	2.9079
ν_{308}	2992.28	56.4851
ν_{309}	2992.31	0.0019
ν_{310}	3003.60	89.9904
ν_{311}	3003.61	1.9103
ν_{312}	3012.45	78.0265
ν_{313}	3012.53	0.0068
ν_{314}	3028.28	3.1120
ν_{315}	3028.28	15.1553
ν_{316}	3033.30	43.8715
ν_{317}	3033.32	0.0014
ν_{318}	3036.61	0.1529
ν_{319}	3036.63	57.4426
ν_{320}	3037.58	0.0192
ν_{321}	3037.58	0.3947
ν_{322}	3040.14	2.9515
ν_{323}	3040.14	36.9910
ν_{324}	3040.58	37.6156
ν_{325}	3040.59	0.0039
ν_{326}	3047.63	83.4934
ν_{327}	3047.66	0.0006
ν_{328}	3050.06	0.0862
ν_{329}	3050.07	150.5472
ν_{330}	3050.95	51.5889
ν_{331}	3050.95	0.0702
ν_{332}	3061.32	8.0287
ν_{333}	3061.32	37.9412
ν_{334}	3063.68	20.2894

ν_{335}	3063.69	0.1106
ν_{336}	3064.94	94.2422
ν_{337}	3064.97	0.0005
ν_{338}	3067.90	69.2967
ν_{339}	3067.91	0.3836
ν_{340}	3070.15	0.0563
ν_{341}	3070.20	210.0481
ν_{342}	3070.64	31.6374
ν_{343}	3070.72	0.0001
ν_{344}	3074.56	3.5803
ν_{345}	3074.56	1.4529
ν_{346}	3075.76	0.0405
ν_{347}	3075.76	24.8872
ν_{348}	3078.85	37.8813
ν_{349}	3078.87	0.1419
ν_{350}	3094.72	1.6349
ν_{351}	3094.72	56.6792
ν_{352}	3102.90	47.8950
ν_{353}	3102.90	0.4821
ν_{354}	3108.14	3.4579
ν_{355}	3108.14	0.0279
ν_{356}	3111.90	0.0033
ν_{357}	3111.91	11.4120
ν_{358}	3120.79	8.3341
ν_{359}	3120.79	34.2243
ν_{360}	3130.78	66.6440

Table S2. Harmonic IR frequencies of CAAC–CS_2 dimer, CAAC–CS_2, and a bis-thiyl diradical (DR) obtained at the B3LYP-GD3/def2-SVP level using a truncated model compound in which the Dipp and ethyl substituents were replaced by methyl groups. Vibrational frequencies were scaled by factors of 0.960, 0.918, and 0.959 for the CAAC–CS_2 dimer, CAAC–CS_2, and DR, respectively.

	CAAC–CS_2 dimer		CAAC–CS_2		Bis-thiyl diradical (DR)	
Vibration mode	Frequency (cm^{-1})	Intensity (km/mol)	Frequency (cm^{-1})	Intensity (km/mol)	Frequency (cm^{-1})	Intensity (km/mol)
ν_{1}	27.69	2.5327	44.69	0.3783	26.12	2.3048
ν_{2}	31.94	3.5394	62.01	1.4959	31.23	1.8125
ν_{3}	58.49	0.0000	75.30	0.5224	60.04	2.8263
ν_{4}	60.81	0.0006	130.03	4.9524	74.35	3.5392
ν_{5}	60.90	3.2988	147.03	5.9841	80.83	4.4068
ν_{6}	78.20	4.6796	157.92	0.6136	87.11	9.0552
ν_{7}	81.43	0.0000	197.53	20.1443	94.59	0.5097
ν_{8}	150.23	0.0000	214.94	2.2233	117.12	1.8841
ν_{9}	155.72	5.6750	240.56	2.9734	132.18	0.9231
ν_{10}	166.16	0.0000	260.75	0.4300	145.70	1.9125
ν_{11}	185.80	2.3816	300.53	1.2199	151.02	13.7656
ν_{12}	187.89	0.0002	315.41	1.4682	160.05	1.0521
ν_{13}	200.41	1.9951	328.49	1.2135	167.95	6.2980
ν_{14}	214.95	0.0000	364.99	1.3395	187.51	4.7043

ν_{15}	218.00	0.0000	406.26	0.5877	198.97	7.1443
ν_{16}	221.87	2.0780	441.71	2.8666	202.83	3.6519
ν_{17}	234.16	0.0000	531.12	3.1203	210.43	18.0354
ν_{18}	241.90	7.4762	549.56	4.3564	213.93	7.4892
ν_{19}	254.32	0.0001	591.99	0.5460	223.76	9.3355
ν_{20}	256.42	2.1661	650.37	13.4332	254.05	2.2836
ν_{21}	264.17	0.0000	724.79	3.5604	256.34	5.1513
ν_{22}	269.69	0.7351	774.24	19.0441	256.69	6.0276
ν_{23}	279.34	0.0003	848.22	31.0212	261.33	9.2408
ν_{24}	280.58	1.8574	868.30	0.6133	280.40	10.7735
ν_{25}	289.78	25.2443	876.43	8.3926	284.62	15.1202
ν_{26}	292.19	0.0006	900.10	9.4146	291.22	4.1052
ν_{27}	306.88	23.4302	917.16	20.6344	298.40	22.3038
ν_{28}	311.15	0.0000	939.67	7.6919	320.71	2.6117
ν_{29}	339.35	0.0000	962.11	16.8335	322.96	11.2986
ν_{30}	340.63	4.2524	972.79	389.3065	343.06	16.0247
ν_{31}	350.03	5.7578	1004.44	42.7823	349.35	0.8564
ν_{32}	356.17	0.0000	1027.42	35.2841	360.82	6.2382
ν_{33}	379.38	0.0000	1069.05	8.6036	379.73	7.1531
ν_{34}	384.60	3.0034	1081.51	16.6873	382.03	4.9184
ν_{35}	411.95	19.0750	1123.90	8.0269	411.82	1.1231
ν_{36}	427.39	0.0000	1132.57	9.2265	417.90	7.3653
ν_{37}	452.64	0.0000	1150.53	7.6949	445.44	17.0377
ν_{38}	463.28	193.0306	1170.27	7.8458	450.41	2.5379
ν_{39}	490.73	48.5281	1208.62	34.7202	492.09	3.3575
ν_{40}	492.91	0.0000	1234.45	7.4581	499.06	25.2566
ν_{41}	553.53	22.1580	1251.50	34.3580	556.68	14.0003
ν_{42}	555.42	0.0000	1274.93	7.6229	558.22	3.8155
ν_{43}	604.32	21.4325	1293.43	6.0185	604.77	21.0805
ν_{44}	604.49	0.0191	1311.24	9.9562	609.52	26.0263
ν_{45}	674.35	72.0626	1327.81	2.8794	620.53	31.9385
ν_{46}	677.30	0.0001	1331.57	8.3196	672.33	85.1344
ν_{47}	747.59	12.6370	1333.78	2.5536	749.68	7.4850
ν_{48}	750.17	0.0000	1341.61	22.7261	758.46	32.9714
ν_{49}	798.98	22.3433	1343.57	10.5400	792.21	35.3101
ν_{50}	801.62	0.0000	1347.52	5.5465	806.78	52.6349
ν_{51}	809.96	35.9420	1356.07	14.6597	842.20	92.0203
ν_{52}	816.07	0.0000	1362.15	7.6744	860.16	167.1231
ν_{53}	896.10	19.3378	1533.04	302.6064	893.03	1.8047
ν_{54}	896.62	0.0009	2787.31	18.7692	895.30	20.2679
ν_{55}	911.16	13.8394	2791.00	18.4132	906.89	32.3509
ν_{56}	911.38	0.0332	2800.76	7.5740	909.09	10.2940
ν_{57}	920.91	3.4632	2804.73	38.7382	920.30	5.1923
ν_{58}	921.29	0.0005	2816.54	18.8865	932.86	62.7947
ν_{59}	950.49	2.6091	2861.91	9.1021	947.41	6.4161
ν_{60}	951.15	0.0000	2866.20	20.5034	954.46	66.7059
ν_{61}	967.81	9.9894	2871.08	21.0760	966.02	23.6275
ν_{62}	968.61	0.0000	2878.11	8.3291	970.99	98.7982
ν_{63}	997.69	1.7029	2882.81	17.2757	995.53	34.7662

ν_{64}	997.81	0.0033	2885.74	2.9960	996.88	0.3327
ν_{65}	1012.91	21.1924	2888.89	13.0225	1005.98	11.2202
ν_{66}	1013.07	0.0215	2921.15	0.5293	1009.22	28.8061
ν_{67}	1053.75	77.9349			1042.65	53.4869
ν_{68}	1054.29	0.0099			1050.52	56.1204
ν_{69}	1076.20	0.0000			1069.34	24.7277
ν_{70}	1076.26	9.6163			1074.33	8.8054
ν_{71}	1125.89	16.0333			1115.17	334.8960
ν_{72}	1125.98	0.0000			1123.36	26.2222
ν_{73}	1141.90	68.2517			1133.95	170.2243
ν_{74}	1142.50	0.0005			1139.75	21.7277
ν_{75}	1173.92	81.1681			1172.53	68.8865
ν_{76}	1174.44	0.0045			1175.19	12.1582
ν_{77}	1192.89	14.7719			1184.79	285.9198
ν_{78}	1192.94	0.0308			1189.11	28.6679
ν_{79}	1229.27	68.2701			1204.14	149.8064
ν_{80}	1229.61	0.0000			1220.27	128.4484
ν_{81}	1237.33	100.5932			1222.86	93.7429
ν_{82}	1238.28	0.0046			1227.17	65.0653
ν_{83}	1262.85	799.3428			1258.31	142.9690
ν_{84}	1268.02	0.0010			1263.31	783.3557
ν_{85}	1300.03	59.0168			1289.30	475.4285
ν_{86}	1300.81	0.0007			1298.42	238.2552
ν_{87}	1330.14	14.8766			1300.78	85.4255
ν_{88}	1330.22	0.0094			1327.36	6.5290
ν_{89}	1344.41	0.0024			1329.31	6.8941
ν_{90}	1344.48	17.9595			1340.17	173.6582
ν_{91}	1367.64	142.8992			1345.32	70.5700
ν_{92}	1368.07	0.0343			1360.24	304.5570
ν_{93}	1379.78	497.8033			1363.70	130.1775
ν_{94}	1384.69	0.0000			1368.69	259.8308
ν_{95}	1387.36	0.0694			1379.50	15.1794
ν_{96}	1387.37	2.8045			1383.34	0.4375
ν_{97}	1392.74	35.7782			1387.18	1.0683
ν_{98}	1392.86	0.0127			1393.87	20.0168
ν_{99}	1401.11	82.4312			1395.69	53.3605
ν_{100}	1401.74	0.0021			1397.58	30.9708
ν_{101}	1404.91	22.7817			1399.01	24.2466
ν_{102}	1405.27	0.0015			1402.21	15.4240
ν_{103}	1411.17	20.5242			1405.03	9.3953
ν_{104}	1411.18	16.2982			1405.91	10.8924
ν_{105}	1413.11	23.8580			1411.80	6.6223
ν_{106}	1413.14	0.0364			1412.63	1.5085
ν_{107}	1422.06	0.0002			1417.07	12.7338
ν_{108}	1422.41	51.6294			1421.33	30.0743
ν_{109}	1433.90	235.2022			1428.70	88.9503
ν_{110}	1435.98	0.0000			1429.74	7.6778
ν_{111}	1498.97	2531.6692			1482.80	1693.8374
ν_{112}	1519.17	0.0002			1520.20	244.5369

ν_{113}	2879.09	260.3943	2882.64	155.2237
ν_{114}	2879.35	0.0022	2903.18	56.8983
ν_{115}	2904.61	0.6412	2907.53	17.8174
ν_{116}	2904.68	120.2264	2909.57	133.1390
ν_{117}	2905.23	129.1526	2909.77	27.3506
ν_{118}	2905.35	0.5417	2911.83	34.3002
ν_{119}	2912.99	62.4951	2914.48	140.9457
ν_{120}	2913.03	0.1323	2917.08	51.6133
ν_{121}	2930.47	36.6465	2930.50	24.6664
ν_{122}	2930.50	11.7977	2949.56	53.3399
ν_{123}	2946.54	118.3306	2950.02	29.8742
ν_{124}	2946.63	9.6350	2960.47	15.9135
ν_{125}	2983.43	29.5933	2981.17	30.6924
ν_{126}	2983.45	34.2947	2985.71	29.3225
ν_{127}	2986.19	0.4903	2986.25	17.3663
ν_{128}	2986.24	29.2730	2988.35	17.1830
ν_{129}	2991.61	1.4837	2990.07	18.7212
ν_{130}	2991.62	10.2189	2992.02	49.1890
ν_{131}	2992.40	131.3184	3000.08	13.7239
ν_{132}	2992.52	0.1454	3003.90	27.2710
ν_{133}	2995.46	27.9338	3005.98	23.9844
ν_{134}	2995.50	38.5660	3017.56	7.6053
ν_{135}	3003.89	45.9056	3025.46	34.7502
ν_{136}	3003.93	1.5739	3026.46	3.9642
ν_{137}	3033.59	0.1450	3030.45	13.8475
ν_{138}	3033.61	28.2265	3065.76	1.9799
